# Association of Pork (All Pork, Fresh Pork and Processed Pork) Consumption with Nutrient Intakes and Adequacy in US Children (Age 2–18 Years) and Adults (Age 19+ Years): NHANES 2011–2018 Analysis

**DOI:** 10.3390/nu15102293

**Published:** 2023-05-13

**Authors:** Sanjiv Agarwal, Victor L. Fulgoni

**Affiliations:** 1 NutriScience LLC, East Norriton, PA 19403, USA; 2 Nutrition Impact, LLC, Battle Creek, MI 49014, USA; vic3rd@aol.com

**Keywords:** pork, National Health and Nutrition Examination Survey, vitamins, minerals, usual intakes, nutrient adequacy

## Abstract

Pork is a rich source of high-quality protein and select nutrients. The objective of this work was to assess the intakes of all pork (AP), fresh pork (FP) and processed pork (PP) and their association with nutrient intake and meeting nutrient recommendations using 24 h dietary recall data. Usual intake was determined using the NCI method and the percentage of the population with intakes below the Estimated Average Requirement, or above the Adequate Intake for pork consumers and non-consumers, was estimated. About 52, 15 and 45% of children and 59, 20 and 49% of adults were consumers of AP, FP and PP, respectively, with mean intakes in consumers of 47, 60 and 38 g/day for children and 61, 77 and 48 g/day for adults, respectively. Among consumers of AP, FP and PP, the intakes of copper, potassium, selenium, sodium, zinc, thiamine, niacin, vitamin B_6_ and choline were higher (*p* < 0.05) and a higher (*p* < 0.05) proportion met nutrient recommendations for copper, potassium, zinc, thiamin and choline compared to non-consumers. There were additional differences (*p* < 0.05) in intakes and adequacies for other nutrients between consumers and non-consumers depending upon the age group and pork type. In conclusion, pork intake was associated with higher intakes and adequacies in children and adults for certain key nutrients.

## 1. Introduction

Pork is one of the most widely consumed meats in the world, accounting for over one-third of meat production and intake globally, and it is a rich source of high-quality protein and select nutrients [1]. The average annual pork consumption in the US is about 51 pounds per person, which is about one-fourth of overall meat intake and ranks third in annual meat consumption [2]. A 100 g portion of pork (pork, not further specified; FDC ID: 2341267) provides substantial amounts of protein (27.1 g, 54.2% DV), iron (0.79 mg, 4.4% DV), zinc (2.44 mg, 22.2% DV), selenium (44.8 µg, 81.5% DV), magnesium (26 mg; 6.2% DV), phosphorus (245 mg; 19.6% DV), potassium (402 mg, 8.6% DV), thiamin (0.605 mg, 50.4% DV), riboflavin (0.234 mg; 18.0% DV), niacin (7.55 mg, 47.2% DV), choline (81.1 mg, 14.7% DV), and vitamins B_6_ (0.615 mg; 36.2% DV) and B_12_ (0.65 µg; 27.1% DV) [3,4]. In cross-sectional analyses, pork consumption has been shown to contribute significantly (more than 10%) to intakes of several nutrients, including protein, phosphorus, potassium, selenium, thiamine, riboflavin, niacin, vitamin B_6_, and vitamin B_12_ [5,6,7], and did not affect diet quality [6]. Limited recent evidence suggests that intake of pork may be associated with cognitive health [8] cardiovascular and metabolic health benefits [9,10,11,12] and reduced risk of functional limitations among older adults [13].

Inadequate micronutrient intakes and deficiencies have been identified as major public health problems affecting a large part of the world’s population and are important contributors to the global burden of disease and increased risk of morbidity and mortality [14,15,16,17,18]. According to recent estimates, 1.5 to 2 billion people, or one-third of the population, suffer from at least one form of micronutrient deficiency [14,15,16,17,18]. Although continued public health recommendations, including the Dietary Guidelines for Americans, suggest consuming nutrient-dense foods as part of healthy eating pattern, many Americans do not adhere to these recommendations and have inadequate intakes of several essential nutrients [19,20]. Therefore, vitamins A, D, E, and C, and choline, calcium, magnesium, iron (for certain age/gender groups), potassium, and fiber have been identified as “under-consumed nutrients” [20] and of these, vitamin D, calcium, iron, potassium, and fiber have been designated as “nutrients of public health concern” because their low intakes may lead to adverse health outcomes and are potentially associated with increased risk of chronic disease [20]. We hypothesize that intake of pork as a rich source of protein and other nutrients would be associated with improved nutrient adequacy for certain nutrients. Therefore, the objective of the present research was to assess the relationship between intake of pork (including fresh pork as well as processed pork) and meeting nutrient recommendations in US children and adults using the National Health and Nutrition Examination Survey (NHANES) 2011–2018.

## 2. Materials and Methods

### 2.1. Database

Dietary intake data from WWEIA component of NHANES 2011–2018 were used in the present analysis. NHANES is an ongoing cross-sectional survey of a nationally representative non-institutionalized civilian population conducted by the National Center for Health Statistics of the Centers for Disease Control and Prevention to monitor food and nutrient intake and the health status of the US population. The data are currently continuously collected using a stratified multistage cluster sampling probability design and are released every 2 years. Participants are interviewed in their homes for demographic, socioeconomic, dietary (24 h dietary recall), and general health information, followed by a comprehensive health examination conducted in a mobile examination center. A detailed description of the subject recruitment, survey design, and data collection procedures is available online [21]. NHANES protocols are approved by the Ethics Review Board of National Center for Health Statistics, and the present study was a secondary data analysis which lacked personal identifiers. Therefore, it was exempt from additional approvals by Institutional Review Boards. All participants provided signed written informed consent. All data obtained from this study are publicly available at: http://www.cdc.gov/nchs/nhanes/, accessed on 12 December 2022.

### 2.2. Study Population

Data from children age 2–18 years (*n* = 10,913; population weighted N = 69,849,814) and adults age 19+ years (*n* = 19,766; population weighted N = 231,605,756) after excluding those with incomplete or unreliable dietary recall as judged by NHANES staff, those with missing day 1 or day 2 dietary data and those pregnant and/or lactating participating in NHANES cycles 2011–2012, 2013–2014, 2015–2016 and 2017–2018 were used.

### 2.3. Estimates of Dietary Intake

Dietary intake data were obtained from in-person 24 h dietary recall interviews that were administered using an automated, multiple-pass (AMPM) method [22]. Nutrient intakes were obtained from the total nutrient intake files for each NHANES cycle [23]; intakes from dietary supplements were not included. Two dietary recalls were collected for most subjects; the first day dietary recall was collected in person, while the second recall was collected via the telephone. The distributions of usual nutrient intakes were estimated using the National Cancer Institute (NCI) method [24] and the percentage of the population below the Estimated Average Requirement (EAR) or above the Adequate Intake (AI) was determined using the cut-point method, except for iron, for which the probability method was used [25].

### 2.4. Estimates of Pork Intakes

The Food and Nutrition Database for Dietary Studies (FNDDS) food codes were used to assess pork intakes by determining the amount of pork contained in NHANES survey foods [26]. When pork items were used as “ingredients” of the survey foods, the FNDDS food codes were identified, and recipe calculations were performed using the survey-specific USDA Food Patterns Equivalents database (FPED) which also includes the Food Patterns Equivalents Ingredient Database (FPID) [26]. The FPID descriptions were examined to determine the proportion of pork: 100% if entirely pork; 50% or 33% if the description indicated one or two other meat types, respectively, in addition to pork. For some FNDDS food codes that contained ingredients with missing FPID, the food code ingredient profile was modified either by using a food code from another NHANES cycle or by using another ingredient code with a similar description. Fresh pork (FP) and processed pork (PP) were defined using the pf_meat and pf_cured meat components, respectively [26]. All pork (AP) included all fresh and processed pork. Consumers of AP, FP, and PP were defined as those individuals consuming any amount of AP, FP, or PP on either of the two days of dietary recall.

### 2.5. Statistics

All analyses were performed using SAS 9.4 (SAS Institute, Cary, NC, USA) software and the data were adjusted for the complex sampling design of NHANES, using appropriate survey weights, strata, and primary sampling units. Day one dietary weights were used in all intake analysis. Data are presented as mean ± standard error; *t*-tests and *z*-statistic was used to assess differences between non-consumers and consumers.

## 3. Results

### 3.1. Children Age 2–18 Years

About 52, 15, and 45% of children age 2-18 years were consumers of AP, FP, and PP, respectively, with a mean intake of 47, 60 and 38 g/day, respectively, among consumers.

**Figure 1:**
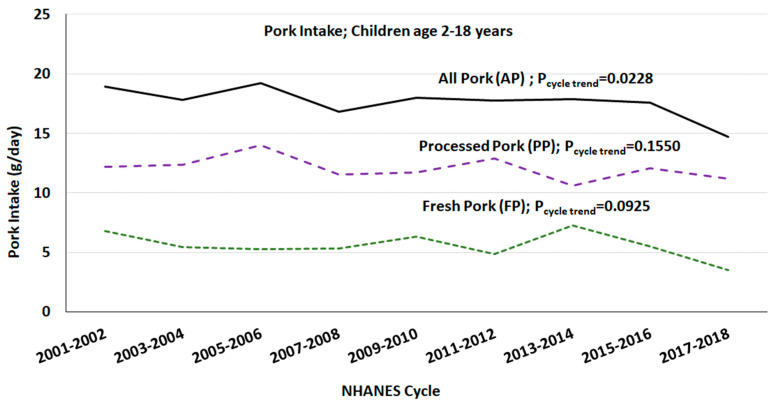
Mean per capita intake of pork among children (age 2–18 years) over NHANES study periods; gender combined data from day one of 24 h dietary recall.

Mean per capita intake of PP, FP and PP were 17, 5 and 12 g/day, respectively. The per capita mean intake of AP has decreased, while intake of FP and PP has not changed over the last 18 years among children age 2-18 years in the US (Figure 1).

Consumers of different pork types (AP, FP, and PP), compared to respective non-consumers, were more likely to be male (except for FP), obese (only for PP), Asian (only for FP), have a poverty–income ratio (PIR) below 1.35 (except for PP), engage in vigorous physical activity (only for FP) and current smokers (only for AP), and less likely to be of normal weight (only for PP), non-Hispanic White (only for FP), Asian (only for AP and PP), and have a PIR above 1.85 (Table 1).

Children who consumed AP, FP, and PP had higher intakes of copper (4–9%), magnesium (4–5%), potassium (7–8%), selenium (13–19%), sodium (5–18%), zinc (5–12%), thiamine (11–13%), niacin (6–9%), vitamin B_6_ (6–7%), and choline (12–19%) compared to their respective non-consumers. Consumers of AP and PP had higher intakes of calcium (5–8%), iron (5–8%), phosphorus (9–11%), riboflavin (7–8%), vitamin B_12_ (9–10%), and vitamin D (7%) than their respective non-consumers. Consumers of PP had higher intakes of folate (5%) than non-consumers. However, consumers of FP had lower intakes for calcium (6%), iron (3%) and vitamin B_12_ (2%) than non-consumers (Table 2).

A higher proportion of children met the nutrient recommendations for copper (3–4% units), potassium (6–10% units), zinc (5–8% units), thiamin (2–3% units), and choline (8–9% units) among consumers of AP, FP, and PP compared to non-consumers. Consumers of AP and PP had lower percentages of children below EAR for calcium (5–8% units), iron (2% units), phosphorus (8–9% units), riboflavin (1–2% units), and vitamin B_12_ (2% units) than non-consumers. Consumers of FP had a lower proportion of children below EAR for magnesium (4% units), vitamin B_6_ (2% units), and vitamin C (11% units) and a higher proportion of children below EAR for calcium (6% units) than non-consumers (Table 3).

### 3.2. Adults Age 19+ Years

About 59, 20, and 49% of adults age 19+ years were consumers of AP, FP, and PP, respectively, with a mean intake of 61, 77, and 48 g/day, respectively, among consumers.

**Figure 2:**
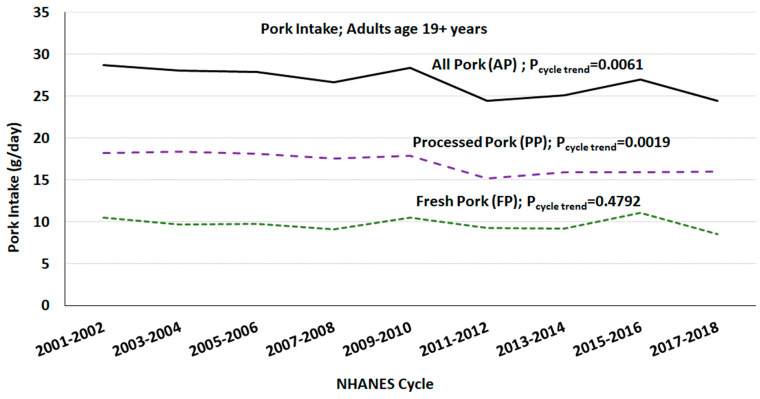
Mean per capita intake of pork among adults (age 19+ years) over NHANES study periods; gender combined data from day one of 24 h dietary recall.

The mean per capita intake of PP, FP and PP was 25, 10, and 16 g/day, respectively. The per capita mean intake of AP and PP has decreased, while the intake of FP has not changed over the last 18 years among those age 19+ years in the US (Figure 2).

Consumers (aged 19+ years) of different pork types (AP, FP, and PP), compared to their respective non-consumers, were more likely to be older, male, obese, non-Hispanic White (only for PP), non-Hispanic Black, Asian (only for FP), have education below High School, High School (except for FP), be sedentary, be a former smoker (only for AP), be current smokers (except for FP), and be less likely to be of normal weight (except for FP), overweight (only for FP), non-Hispanic White (only for FP), Asian (except for FP), have education above High School, engage in vigorous activity (except for PP), and be never smokers (Table 4).

Adult consumers of AP, FP, and PP had higher intakes of iron (3–6%), phosphorus (3–12%), potassium (6–8%), selenium (15–19%), sodium (7–20%), zinc (8–11%), thiamine (14–20%), riboflavin (1–10%), niacin (9–11%), vitamin B_6_ (4–6%), and choline (13–21%) compared to their respective non-consumers. Consumers of AP and PP had higher intakes of calcium (5–11%) and vitamin B_12_ (6–8%) than their respective non-consumers. Consumers of PP had higher intakes of vitamin D (6%). However, consumers of AP had lower intakes of vitamin C (5%), and consumers of FP had lower intakes of calcium (9%), vitamin A (9%) and vitamin E (4%) compared to their respective non-consumers (Table 5).

A higher proportion of adults met the nutrients recommendations for copper (2–5% units), iron (2–4% units), phosphorus (~1% unit), potassium (4–5% units), selenium (1% units), sodium (1–2% units), zinc (5–12% units), thiamin (8–12% units), riboflavin (1–4% units), niacin (1–3% units), vitamin B_6_ (5–6% units), and choline (5–6% units) among consumers of AP, FP, and PP compared to their respective non-consumers. Consumers of AP and PP had lower proportion of adults below EAR for calcium (6–11% units), folate (3–4% units), and vitamin B_12_ (5% units) than their respective non-consumers. Consumers of PP had lower proportion of adults below EAR vitamin A (5% units) than non-consumers. However, consumers of AP and PP had a higher proportion of adults below EAR for vitamin D (2–3% units); consumers of AP and FP had a higher proportion of adults below EAR for vitamin E (4–5% units), and consumers of FP had a higher proportion of adults below EAR for calcium (8% units) and vitamin A (8% units) compared to their respective non-consumers (Table 6).

## 4. Discussion

The results of this analysis of cross-sectional data from NHANES indicate that children and adult consumers of pork have higher intakes and lower prevalence of inadequacies of several key micronutrients, including many under-consumed nutrients and nutrients of concern, compared to those who did not consume pork. Interestingly, the results for most nutrients are similar for fresh pork and processed pork consumers.

To date, only a limited number of studies have evaluated the impact of pork intake on micronutrient intakes, and even fewer have assessed the association of pork intake with meting nutrient recommendations. In an earlier cross-sectional analysis of NHANES 2003–2006, Murphy et al. [5] reported that fresh pork and fresh lean pork contributed more than 10% of daily intake of protein, phosphorus, potassium, zinc, selenium, thiamine, riboflavin, niacin, vitamin B_6_, and vitamin B_12_ in the diets of consumers. Increased fresh and lean pork intakes were related to small but significantly (*p* < 0.01) improved daily nutritional intakes of protein (4 g), magnesium (4 mg), phosphorus (30 mg), potassium (83–85 mg), selenium (7 µg), zinc (0.3 mg), thiamine (0.2 mg), riboflavin (0.04 mg), niacin (0.8 mg), and vitamin B_6_ (0.1 mg) compared to non-consumers in another cross-sectional analysis of NHANES 2005–2016 [6]. In a secondary analysis of the 2007 Australian National Children’s Nutrition and Physical Activity Survey [7], fresh pork contributed substantially to the total intakes of thiamin (15%), protein (13%), niacin (10%), zinc (9%), phosphorous (7%), and potassium (6%); while processed pork contributed protein (6.3%), zinc (5.4%), and niacin (5.2%), in the diets of children. In the present analysis of NHANES 2011–2018, we find that both children and adult consumers of different pork types had consistently significantly higher intakes of several micronutrients, including potassium, selenium, zinc, thiamine, niacin, vitamin B_6_, and choline, compared to their respective non-consumers. Consumers of one or the other pork types also had significantly higher intakes of several other micronutrients. Interestingly, calcium intakes were higher among consumers of both AP and PP but lower among consumers of FP. The reasons for this anomaly are not immediately apparent and require further investigation, but we hypothesize that consumers of pork may also consume more calcium-rich dairy products.

In addition to higher intakes, consumers of different pork types also had a lower prevalence of % population below EAR and higher prevalence of % population above AI compared to non-consumers for several nutrients. To the best of our knowledge, ours is this first investigation on different types of pork intakes and meeting nutrient recommendations. However, many of the observed differences in the prevalence of nutritional inadequacies (% population below EAR) or % population above AI between consumers and non-consumers of different types of pork were in mid-single digits for both children and adults (see Table 3 and Table 5). To put these results into perspective, since we used population weighted nationally representative data, a sample size of 5757 children and 11,555 adult consumers of AP represented 36,523,218 children and 135,707,272 adult consumers of AP; a 1% unit change in prevalence of meeting nutritional requirement among consumers would translate to 365,232 children and 1,357,072 adults. For example, based on our results 7.09% more children and 4.25% more adult consumers of AP being above the AI for potassium, we estimate that pork (AP) intake was associated with over 2.5 million more children and over 5.7 million more adults meeting the adequate intake level of potassium.

There is a consistent ongoing global debate on the climate and other environmental effects of animal agriculture and animal sourced food production while ensuring food security for the growing populations. Many scientists and policy makers are increasingly concerned with the environmental consequences in addition to the potential health consequences of meat (especially red meat) consumption and have advocated to limit or eliminate animal-sourced food from the diet [27,28,29,30]. However, such recommendations that primarily account for the environmental impact of animal sourced foods do not necessarily account for their potential effect on food availability and nutrient intake and could have potential unintended consequences [31,32,33]. However, pork production has been shown to be associated with greenhouse gas emission to a lesser extent compared to ruminant meat [34], and therefore would have less environmental impact.

The major strengths of our study included the use of a large nationally representative, population-based sample achieved through combining several sets of NHANES data releases and the use of the NCI method to assess usual intake to assess the percentage of the population below the EAR or above the AI. A major limitation of the current study, as with any cross-sectional study, is the inability to determine the cause-and-effect relationship. Additionally, there is the potential for bias in the use of self-reported dietary recalls relying on memory [35].

Finally, our findings suggest several future research opportunities: (1) while we looked at broad age groups to determine the overall impact of pork, there may be value in further evaluation of the association of pork intake in diverse groups based on age, socioeconomic status, and race/ethnicity; (2) if possible, an evaluation of the impact of specific pork cuts/parts of the pig may be worthwhile; (3) given the different base diets around the world, it may be worthwhile to evaluate the impact of pork in different parts of the world based on geography/cultural background; and (4) modeling to help define what foods would need to be consumed in greater quantities to replace nutrients from pork if removed from the diet.

## 5. Conclusions

The results show that pork intake was associated with improved nutrient intake and meeting nutrient recommendations in US children (age 2–18 years) and adults (age 19+ years) for certain key nutrients. It is therefore likely that pork may play a critical role in reducing the incidence of under-nutrition. At a minimum, those that advocate removal of meat and, in particular, pork from dietary guidelines need to ensure the nutrients provided by pork are replaced with other dietary changes. Future studies are needed to examine the long-term impact of pork consumption on diet quality, nutrient intake, and health promotion.

## Figures and Tables

**Table 1:** Demographics associated with pork consumption in children (age 2–18 years).

	All Pork (AP)		Fresh Pork (FP)		Processed Pork (PP)	
	Non-		Non-		Non-	
	Consumers	Consumers	Consumers	Consumers	Consumers	Consumers
Sample N	5156	5757	9077	1836	6103	4810
Population N	33,326,596	36,523,218	59,684,896	10,164,918	38,532,690	31,317,124
Mean Age (Years)	9.94 ± 0.13	10.2 ± 0.1	10.1 ± 0.1	9.95 ± 0.17	9.94 ± 0.11	10.3 ± 0.1
Gender (% Male)	48.9 ± 1.1	52.6 ± 1.0 ^#^	50.7 ± 0.9	51.7 ± 1.5	49.2 ± 1.0	52.8 ± 1.1 ^#^
Underweight (%)	4.07 ± 0.52	3.32 ± 0.42	3.76 ± 0.38	3.16 ± 0.60	4.00 ± 0.51	3.28 ± 0.47
Normal weight (%)	63.1 ± 1.1	60.8 ± 1.0	62.1 ± 0.8	60.5 ± 1.6	63.1 ± 1.0	60.3 ± 1.1 ^#^
Overweight (%)	15.2 ± 0.7	16.4 ± 0.7	15.8 ± 0.5	16.3 ± 1.0	15.3 ± 0.6	16.5 ± 0.7
Obese (%)	17.6 ± 0.9	19.5 ± 0.9	18.4 ± 0.8	20.0 ± 1.2	17.6 ± 0.8	19.9 ± 1.0 ^#^
Ethnicity						
Hispanic (%)	22.9 ± 1.9	24.8 ± 2.1	23.8 ± 1.9	24.3 ± 2.7	22.9 ± 1.9	25.2 ± 2.2
n-H White (%)	52.9 ± 2.5	51.0 ± 2.7	53.0 ± 2.5	45.5 ± 3.2 *	52.1 ± 2.5	51.6 ± 2.8
n-H Black (%)	13.0 ± 1.3	14.5 ± 1.5	13.4 ± 1.3	16.2 ± 2.1	13.3 ± 1.3	14.4 ± 1.6
Asian (%)	5.33 ± 0.62	4.06 ± 0.43 ^#^	3.99 ± 0.43	8.66 ± 1.05 *	5.94 ± 0.62	3.11 ± 0.38 *
Poverty Income Ratio						
<1.35 (%)	33.7 ± 1.8	36.7 ± 1.8 ^#^	34.5 ± 1.7	39.9 ± 2.6 ^#^	34.2 ± 1.8	36.5 ± 1.8
1.35 ≤ 1.85 (%)	11.1 ± 0.8	12.0 ± 0.8	11.3 ± 0.7	12.7 ± 1.4	11.3 ± 0.8	11.8 ± 0.9
>1.85 (%)	55.2 ± 2.0	51.4 ± 1.9 *	54.2 ± 1.9	47.4 ± 2.2 *	54.5 ± 1.9	51.6 ± 2.0 ^#^
Education						
<High School (%)	99.3 ± 0.2	99.3 ± 0.2	99.2 ± 0.1	99.7 ± 0.2	99.3 ± 0.2	99.2 ± 0.2
High school (%)	0.73 ± 0.16	0.71 ± 0.18	0.78 ± 0.14	0.32 ± 0.18	0.69 ± 0.15	0.75 ± 0.21
>High School (%)	0.00 ± 0.00	0.00 ± 0.00	0.00 ± 0.00	0.00 ± 0.00	0.00 ± 0.00	0.00 ± 0.000
Physical Activity						
Sedentary (%)	15.6 ±0.9	14.9 ± 0.8	15.5 ± 0.7	13.9 ± 1.1	15.4 ± 0.9	15.1 ± 0.9
Moderate (%)	24.1 ± 1.0	24.9 ± 0.7	24.9 ± 0.7	22.5 ± 1.6	23.9 ± 0.9	25.3 ± 0.9
Vigorous (%)	60.4 ± 1.2	60.1 ± 1.1	59.7 ± 1.0	63.6 ± 1.8 ^#^	60.7 ± 1.2	59.6 ± 1.2
Smoking never (%)	92.8 ± 0.6	91.6 ± 0.6	92.3 ± 0.5	91.3 ± 1.2	92.7 ± 0.6	91.5 ± 0.7
Smoking former (%)	6.04 ± 0.56	6.70 ± 0.53	6.30 ± 0.43	6.90 ± 1.03	6.11 ± 0.54	6.73 ± 0.60
Smoking current (%)	0.80 ± 0.19	1.60 ± 0.31 ^#^	1.16 ± 0.22	1.58 ± 0.68	0.86 ± 0.19	1.65 ± 0.38

Two days 24 h dietary recall data from NHANES 2011–2018. Data is presented as Mean ± Standard Error. ^#^, * represent significant differences from non-consumers at *p* < 0.05 and *p* < 0.01, respectively and assessed by *t*-tests. n-H, non-Hispanic.

**Table 2:** Usual intakes of nutrients among children (age 2–18 years, gender combined) non-consumers and consumers of different pork types.

	All Pork (AP)		Fresh Pork (FP)		Processed Pork (PP)	
	Non-		Non-		Non-	
	Consumers	Consumers	Consumers	Consumers	Consumers	Consumers
Sample N	5156	5757	9077	1836	6103	4810
Population N	33,326,596	36,523,218	59,684,896	10,164,918	38,532,690	31,317,124
Calcium (mg)	983 ± 14	1035 ± 11 *	1018 ± 11	954 ± 15 *	976 ± 12	1052 ± 12 *
Copper (mg)	0.89 ± 0.01	0.96 ± 0.01 *	0.92 ± 0.01	0.96 ± 0.02 ^#^	0.89 ± 0.01	0.97 ±0.01 *
Iron (mg)	13.4 ± 0.2	14.1 ± 0.2 *	13.8 ± 0.1	13.4 ± 0.2	13.3 ± 0.2	14.3 ± 0.2 *
Magnesium (mg)	228 ± 2	238 ± 2 *	232 ± 2	241 ± 4 ^#^	228 ± 2	239 ± 3 *
Phosphorus (mg)	1197 ± 13	1304 ± 13 *	1252 ± 10	1271 ± 19	1198 ± 11	1324 ± 14 *
Potassium (mg)	2055 ± 21	2217 ± 23 *	2116 ± 16	2295 ± 38 *	2073 ± 20	2224 ± 25 *
Selenium (µg)	86 ± 1.0	102 ± 1 *	92.7 ± 0.8	105 ± 2 *	87.7 ± 0.9	103 ± 1 *
Sodium (mg)	2723 ± 29	3185 ± 34 *	2940 ± 26	3095 ± 57 ^#^	2737 ± 26	3240 ± 35 *
Zinc (mg)	9.18 ± 0.12	10.3 ± 0.1 *	9.68 ± 0.10	10.2 ± 0.2 ^#^	9.28 ± 0.12	10.4 ± 0.1 *
Vitamin A, RE (µg)	586 ± 9	604 ± 9	600 ± 8	579 ± 11	585 ± 8	609 ± 10
Thiamin (mg)	1.43 ± 0.02	1.62 ± 0.02 *	1.5 ± 0.01	1.67 ± 0.03 *	1.45 ± 0.02	1.63 ± 0.02 *
Riboflavin (mg)	1.84 ± 0.02	1.97 ± 0.02 *	1.90 ± 0.02	1.94 ± 0.03	1.84 ± 0.02	1.98 ± 0.02 *
Niacin (mg)	20.3 ± 0.3	22.2 ± 0.3 *	21.1 ± 0.2	22.4 ± 0.4 *	20.5 ± 0.2	22.3 ± 0.4 *
Folate, DFE (µg)	495 ± 8	512 ± 8	507 ± 6	487 ± 10	492 ± 7	516 ± 9 ^#^
Vitamin B_6_ (mg)	1.66 ± 0.02	1.78 ± 0.03 *	1.70 ± 0.02	1.81 ± 0.04 ^#^	1.67 ± 0.02	1.78 ± 0.03 *
Vitamin B_12_ (µg)	4.45 ± 0.08	4.85 ± 0.08 *	4.67 ± 0.06	4.58 ± 0.09	4.46 ± 0.07	4.89 ± 0.09 *
Vitamin C (mg)	71.2 ± 1.7	76 ± 2.1	72.5 ± 1.5	81.5 ± 5.0	71.6 ± 1.6	76.4 ± 2.2
Vitamin D (µg)	5.20 ± 0.12	5.54 ± 0.09 ^#^	5.36 ± 0.09	5.45 ± 0.14	5.23 ± 0.11	5.57 ± 0.10 ^#^
Vitamin E, ATE (mg)	7.08 ± 0.13	7.26 ± 0.10	7.16 ± 0.09	7.20 ± 0.16	7.05 ± 0.11	7.30 ± 0.11
Choline (mg)	226 ± 3	269 ± 3 *	244 ± 2	274 ± 5 *	229 ± 3	273 ± 3 *

Two days 24 h dietary recall data from NHANES 2011–2018. Data presented as mean ± Standard Error; ATE: alpha tocopherol equivalents; DFE: dietary folate equivalents; RE: retinol activity equivalents; ^#^,* represent significant differences from non-consumers at *p* < 0.05 and *p* < 0.01, respectively and assessed by *z* statistics.

**Table 3:** Nutrient inadequacy/adequacy in children (age 2–18 years, gender combined) non-consumers and consumers of different pork types.

	All Pork (AP)		Fresh Pork (FP)		Processed Pork (PP)	
	Non-		Non-		Non-	
	Consumers	Consumers	Consumers	Consumers	Consumers	Consumers
Sample N	5156	5757	9077	1836	6103	4810
Population N	33,326,596	36,523,218	59,684,896	10,164,918	38,532,690	31,317,124
% Children below Estimated Average Requirements (EAR)						
Calcium	48.8 ± 1.5	44.0 ± 1.3 ^#^	45.7 ± 1.4	52.0 ± 1.7 *	49.6 ± 1.4	42.0 ± 1.5 *
Copper	7.05 ± 0.84	3.32 ± 0.59 *	5.52 ± 0.59	2.08 ± 0.59 *	6.73 ± 0.75	3.06 ± 0.66 *
Iron	4.03 ± 0.54	2.15 ± 0.37 *	3.01 ± 0.39	2.54 ± 0.61	4.03 ± 0.49	1.89 ± 0.41 *
Magnesium	36.6 ± 1.3	34.0 ± 1.2	35.6 ± 1.0	31.7 ± 1.6 ^#^	36.1 ± 1.1	34.0 ± 1.3
Phosphorus	19.7 ± 1.4	11.3 ± 1.2 *	15.6 ± 1.1	11.8 ± 1.7	19.4 ± 1.2	10.1 ± 1.3 *
Selenium	0.16 ± 0.08	0.02 ± 0.02	0.11 ± 0.05	0.0004 ± 0.01 ^#^	0.10 ± 0.06	0.02 ± 0.03
Zinc	15.1 ± 1.6	7.58 ± 1.22 *	11.7 ± 1.2	6.4 ± 1.5 *	14.6 ± 1.4	6.82 ± 1.43 *
Vitamin A	26.0 ± 1.5	23.8 ± 1.7	24.8 ± 1.3	25.2 ± 2.1	26.2 ± 1.3	23.31 ± 1.95
Thiamin	3.14 ± 0.58	0.62 ± 0.29 *	2.02 ± 0.41	0.12 ± 0.12 *	2.78 ± 0.50	0.62 ± 0.34 *
Riboflavin	2.06 ± 0.45	0.68 ± 0.27 *	1.28 ± 0.35	0.73 ± 0.26	2.10 ± 0.43	0.54 ± 0.27 *
Niacin	0.65 ± 0.23	0.17 ± 0.11	0.42 ± 0.15	0.05 ± 0.06 ^#^	0.53 ± 0.19	0.16 ± 0.13
Folate	5.39 ± 0.84	3.81 ± 0.88	4.47 ± 0.83	4.26 ± 1.23	5.44 ± 0.77	3.53 ± 1.04
Vitamin B_6_	3.33 ± 0.74	1.66 ± 0.60	2.74 ± 0.61	0.76 ± 0.48 ^#^	3.16 ± 0.69	1.61 ± 0.66
Vitamin B_12_	2.41 ± 0.46	0.87 ± 0.33 *	1.65 ± 0.36	0.81 ± 0.37	2.31 ± 0.42	0.78 ± 0.34 *
Vitamin C	23.1 ± 1.3	21.3 ± 1.8	23.7 ± 1.1	13.0 ± 4.2 ^#^	22.5 ± 1.3	21.8 ± 1.9
Vitamin D	93.1 ± 0.8	93.9 ± 0.7	93.3 ± 0.6	94.2 ± 1.2	93.0 ± 0.8	94.1 ± 0.8
Vitamin E	66.1 ± 1.7	65.9 ± 1.1	66.1 ± 1.2	64.6 ± 2.1	66.2 ± 1.5	65.8 ± 1.3
% Children above Adequate Intake (AI)						
Potassium	27.3 ± 1.5	34.4 ± 1.4 *	29.6 ± 1.1	39.8 ± 2.6 *	28.2 ± 1.3	34.4 ± 1.6 *
Sodium	99.7 ± 0.1	100 ± 0.02 ^#^	99.8 ± 0.1	100 ± 0.02 ^#^	99.7 ± 0.1	100 ± 0.02 ^#^
Choline	16.1 ± 1.1	25.3 ± 0.9 *	19.7 ± 0.8	27.6 ± 1.7 *	16.7 ± 1.0	26.1 ± 1.0 *

Two days 24 h dietary recall data from NHANES 2011–2018. Data presented as mean ± Standard Error; ^#^,* represent significant differences from non-consumers at *p* < 0.05 and *p* < 0.01, respectively and assessed using *z* statistics.

**Table 4:** Demographics associated with pork consumption in adults (age 19+ years).

	All Pork (AP)		Fresh Pork (FP)		Processed Pork (PP)	
	Non-		Non-		Non-	
	Consumers	Consumers	Consumers	Consumers	Consumers	Consumers
Sample N	8211	11,555	15,367	4399	10,340	9426
Population N	95,898,484	135,707,272	184,749,722	46,856,034	118,070,955	113,534,800
Mean Age (Years)	46.5 ± 0.4	48.5 ± 0.4 *	47.4 ± 0.3	48.6 ± 0.6 *	47.0 ± 0.4	48.3 ± 0.4 *
Gender (% Male)	44.8 ± 0.8	52.6 ± 0.6 *	48.4 ± 0.6	53.2 ± 1.0 *	45.8 ± 0.7	53.1 ± 0.7 *
Underweight (%)	1.61 ± 0.23	1.48 ± 0.16	1.54 ± 0.15	1.51 ± 0.24	1.69 ± 0.22	1.37 ± 0.17
Normal weight (%)	30.6 ± 1.0	25.3 ± 0.8 *	27.6 ± 0.7	27.0 ± 1.0	30.2 ± 0.9	24.7 ± 0.9 *
Overweight (%)	32.4 ± 0.9	32.2 ± 0.8	33.0 ± 0.6	29.2 ± 1.1 *	31.5 ± 0.8	33.0 ± 0.9
Obese (%)	35.4 ± 1.0	41.1 ± 0.8 *	37.8 ± 0.8	42.3 ± 1.0 *	36.7 ± 0.9	40.9 ± 0.9 *
Ethnicity						
Hispanic (%)	15.4 ± 1.2	14.8 ± 1.2	15.0 ± 1.2	15.3 ± 1.4	15.3 ± 1.2	14.8 ± 1.3
n-H White (%)	63.4 ± 1.7	65.4 ± 1.9	65.7 ± 1.6	59.8 ± 2.3 *	62.4 ± 1.8	66.8 ± 1.8 *
n-H Black (%)	10.6 ± 0.9	12.0 ± 1.1 ^#^	11.0 ± 0.9	13.1 ± 1.4 ^#^	10.7 ± 0.9	12.1 ± 1.1 ^#^
Asian (%)	6.75 ± 0.62	4.67 ± 0.48 *	4.61 ± 0.42	9.17 ± 0.98 *	7.97 ± 0.71	3.00 ± 0.31 *
Poverty Income Ratio						
<1.35 (%)	24.4 ± 1.0	24.2 ± 1.0	24.1 ± 1.0	24.9 ± 1.5	24.4 ± 1.0	24.1 ± 1.1
1.35 ≤ 1.85 (%)	10.3 ± 0.7	9.71 ± 0.48	9.84 ± 0.46	10.3 ± 0.8	10.4 ± 0.6	9.45 ± 0.50
>1.85 (%)	65.4 ± 1.4	66.1 ± 1.3	66.1 ± 1.2	64.8 ± 1.9	65.2 ± 1.4	66.4 ± 1.3
Education						
<High School (%)	34.9 ± 1.4	38.9 ± 1.1 *	36.4 ± 1.2	40.5 ± 1.4 *	35.9 ± 1.2	38.6 ± 1.2 *
High School (%)	31.7 ± 1.0	33.4 ± 0.7 ^#^	32.8 ± 0.7	32.5 ± 1.1	31.4 ± 0.8	34.0 ± 0.8 *
>High School (%)	33.4 ± 1.4	27.7 ± 1.3 *	30.8 ± 1.3	27.1 ± 1.6 *	32.6 ± 1.3	27.4 ± 1.3 *
Physical Activity						
Sedentary (%)	19.7 ± 0.6	22.6 ± 0.8 *	20.9 ± 0.6	23.3 ± 1.0 ^#^	20.4 ± 0.6	22.3 ± 0.8 ^#^
Moderate (%)	35.8 ± 0.9	35.7 ± 0.8	35.4 ± 0.7	37.1 ± 1.2	36.0 ± 0.8	35.5 ± 0.9
Vigorous (%)	44.5 ± 0.9	41.7 ± 1.0 ^#^	43.7 ± 0.8	39.6 ± 1.2 *	43.6 ± 0.9	42.1 ± 1.0
Smoking never (%)	57.2 ± 0.9	52.4 ± 0.8 *	55.1 ± 0.7	51.7 ± 1.2 *	56.5 ± 0.9	52.2 ± 0.8 *
Smoking former (%)	25.3 ± 0.9	27.5 ± 0.6 ^#^	26.1 ± 0.6	28.3 ± 1.1	25.7 ± 0.8	27.5 ± 0.7
Smoking current (%)	17.3 ± 0.8	19.9 ± 0.7 *	18.5 ± 0.7	19.8 ± 1.1	17.6 ± 0.8	20.0 ± 0.8 *

Two days 24 h dietary recall data from NHANES 2011–2018. Data are presented as Mean ± Standard Error. ^#^,* represent significant differences from non-consumers at *p* < 0.05 and *p* < 0.01, respectively, and assessed using *t*-tests. n-H: non-Hispanic.

**Table 5:** Usual intakes of nutrients among adults (age 19+ years, gender combined) non-consumers and consumers of different pork types.

	All Pork (AP)		Fresh Pork (FP)		Processed Pork (PP)	
	Non-		Non-		Non-	
	Consumers	Consumers	Consumers	Consumers	Consumers	Consumers
Sample N	8211	11,555	15,367	4399	10,340	9426
Population N	95,898,484	135,707,272	184,749,722	46,856,034	118,070,955	113,534,800
Calcium (mg)	944 ± 10	987 ± 8 *	987 ± 6	902 ± 11 *	922 ± 8	1019 ± 9 *
Copper (mg)	1.24 ± 0.02	1.27 ± 0.01	1.25 ± 0.01	1.28 ± 0.01	1.24 ± 0.01	1.27 ± 0.01
Iron (mg)	14.2 ± 0.2	15 ± 0.1 *	14.6 ± 0.1	15 ± 0.2 ^#^	14.3 ± 0.1	15.0 ± 0.1 *
Magnesium (mg)	306 ± 3	309 ± 2	307 ± 2	312 ± 3	306 ± 3	309 ± 2
Phosphorus (mg)	1317 ± 13	1457 ± 8 *	1391 ± 7	1432 ± 13 *	1320 ± 11	1481 ± 9 *
Potassium (mg)	2558 ± 25	2756 ± 19 *	2643 ± 17	2803 ± 33 *	2591 ± 21	2769 ± 21 *
Selenium (µg)	104 ± 1	124 ± 1 *	112 ± 1	130 ± 1 *	108 ± 1	124 ± 1 *
Sodium (mg)	3171 ± 25	3812 ± 23 *	3495 ± 17	3757 ± 34 *	3221 ± 22	3881 ± 24 *
Zinc (mg)	10.6 ± 0.1	11.8 ± 0.1 *	11.1 ± 0.1	12.0 ± 0.1 *	10.7 ± 0.1	11.8 ± 0.1 *
Vitamin A, RE (µg)	652 ± 13	637 ± 10	656 ± 9	595 ± 16 *	636 ± 11	652 ± 10
Thiamin (mg)	1.46 ± 0.01	1.73 ± 0.01 *	1.55 ± 0.01	1.86 ± 0.02 *	1.51 ± 0.01	1.72 ± 0.01 *
Riboflavin (mg)	2.05 ± 0.02	2.23 ± 0.02 *	2.15 ± 0.02	2.18 ± 0.02 *	2.06 ± 0.02	2.26 ± 0.02 *
Niacin (mg)	24.7 ± 0.3	27.4 ± 0.2 *	25.8 ± 0.2	28.0 ± 0.3 *	25.1 ± 0.2	27.5 ± 0.2 *
Folate, DFE (µg)	523 ± 7	533 ± 4	529 ± 4	534 ± 7	524 ± 6	534 ± 5
Vitamin B_6_ (mg)	2.10 ± 0.03	2.21 ± 0.02 *	2.14 ± 0.02	2.27 ± 0.03 *	2.12 ± 0.02	2.21 ± 0.02 *
Vitamin B_12_ (µg)	4.85 ± 0.08	5.16 ± 0.07 *	5.05 ± 0.06	4.95 ± 0.11	4.83 ± 0.07	5.23 ± 0.08 *
Vitamin C (mg)	82.2 ± 1.6	78.0 ± 1.3 ^#^	79.7 ± 1.2	79.9 ± 1.9	81.6 ± 1.5	77.7 ± 1.3
Vitamin D (µg)	4.48 ± 0.09	4.64 ± 0.07	4.56 ± 0.06	4.58 ± 0.13	4.45 ± 0.09	4.71 ± 0.8 ^#^
Vitamin E, ATE (mg)	9.32 ± 0.15	9.21 ± 0.10	9.35 ± 0.11	8.94 ± 0.12 ^#^	9.15 ± 0.13	9.37 ± 0.11
Choline (mg)	299 ± 3	362 ± 3 *	328 ± 2	369 ± 5 *	307 ± 3	366 ± 3 *

Two days 24 h dietary recall data from NHANES 2011–2018. Data presented as mean ± Standard Error; ATE: alpha tocopherol equivalents; DFE: dietary folate equivalents; RE: retinol activity equivalents; ^#^,* represent significant differences from non-consumers at *p* < 0.05 and *p* < 0.01, respectively, and assessed using *z* statistics.

**Table 6:** Nutrient inadequacy/adequacy in adult (age 19+ years, gender combined) non-consumers and consumers of different pork types.

	All Pork (AP)		Fresh Pork (FP)		Processed Pork (PP)	
	Non-		Non-		Non-	
	Consumers	Consumers	Consumers	Consumers	Consumers	Consumers
Sample N	8211	11,555	15,367	4399	10,340	9426
Population N	95,898,484	135,707,272	184,749,722	46,856,034	118,070,955	113,534,800
% Adults below Estimated Average Requirements (EAR)						
Calcium	46.9 ± 1.1	41.1 ± 1.0 *	41.9 ± 0.7	49.9 ± 1.2 *	49.0 ± 0.9	37.6 ± 1.0 *
Copper	10.7 ± 0.6	5.58 ± 0.46 *	8.12 ± 0.48	6.11 ± 0.75 ^#^	10.1 ± 0.5	5.20 ± 0.47 *
Iron	7.58 ± 0.40	4.03 ± 0.22 *	5.75 ± 0.26	4.00 ± 0.43 *	7.01 ± 0.34	3.91 ± 0.25 *
Magnesium	52.2 ± 1.3	53.1 ± 0.9	52.6 ± 0.9	52.4 ± 1.3	52.4 ± 1.0	53.1 ± 1.0
Phosphorus	1.77 ± 0.25	0.32 ± 0.05 *	0.87 ± 0.11	0.52 ± 0.12 ^#^	1.68 ± 0.23	0.23 ± 0.06 *
Selenium	1.46 ± 0.30	0.13 ± 0.04 *	0.75 ± 0.11	0.09 ± 0.05 *	1.28 ± 0.24	0.12 ± 0.04 *
Zinc	24.6 ± 1.2	12.6 ± 1.0 *	18.9 ± 0.9	13.6 ± 1.4 *	23.4 ± 1.1	11.7 ± 1.2 *
Vitamin A	44.9 ± 1.3	44.7 ± 1.5	43.1 ± 1.1	51.2 ± 2.5 *	46.6 ± 1.2	42.0 ± 1.6 ^#^
Thiamin	14.6 ± 0.9	2.98 ± 0.38 *	9.42 ± 0.54	1.04 ± 0.36 *	12.0 ± 0.8	3.06 ± 0.44 *
Riboflavin	5.66 ± 0.56	2.23 ± 0.23 *	3.83 ± 0.29	2.50 ± 0.42 *	5.38 ± 0.50	1.86 ± 0.23 *
Niacin	3.25 ± 0.48	0.73 ± 0.13 *	1.93 ± 0.22	0.65 ± 0.22 *	2.71 ± 0.38	0.65 ± 0.15 *
Folate	15.6 ± 1.0	12.4 ± 0.9 ^#^	14.0 ± 0.7	13.2 ± 1.5	15.5 ± 1.0	11.9 ± 1.0 *
Vitamin B_6_	15.1 ± 1.1	9.45 ± 0.73 *	13.0 ± 0.8	8.08 ± 1.08 *	14.2 ± 0.9	9.48 ± 0.87 *
Vitamin B_12_	8.34 ± 0.96	3.26 ± 0.47 *	5.88 ± 0.55	3.93 ± 0.83	8.16 ± 0.75	2.74 ± 0.47 *
Vitamin C	47.2 ± 1.3	49.5 ± 1.1	48.6 ± 1.1	47.8 ± 1.7	47.1 ± 1.2	49.8 ± 1.2
Vitamin D	93.8 ± 0.5	96.5 ± 0.4 *	95.0 ± 0.4	96.2 ± 0.7	94.3 ± 0.5	96.4 ± 0.4 *
Vitamin E	76.8 ± 1.3	81.0 ± 0.8 *	78.0 ± 1.0	83.2 ± 1.1 *	78.5 ± 1.0	79.9 ± 1.0
% Adults above Adequate Intake (AI)						
Potassium	29.2 ± 1.2	33.4 ± 1.0 *	30.8 ± 0.9	35.8 ± 1.7 ^#^	30.1 ± 1.1	33.9 ± 1.1 ^#^
Sodium	98.2 ± 0.4	99.8 ± 0.07 *	99.1 ± 0.1	99.6 ± 0.13 *	98.4 ± 0.3	99.8 ± 0.1 *
Choline	4.43 ± 0.53	10.6 ± 0.9 *	7.33 ± 0.54	12.1 ± 1.3 *	5.15 ± 0.55	11.0 ± 1.0 *

Two days 24 h dietary recall data from NHANES 2011–2018. Data presented as mean ± Standard Error; ^#^,* represent significant differences from non-consumers at *p* < 0.05 and *p* < 0.01, respectively and assessed using *z* statistics.

## Data Availability

The datasets analyzed in this study are available in the Center for Disease Control and Prevention repository; available online: http://www.cdc.gov/nchs/nhanes/ (accessed on 12 December 2022).
